# Identification of a biliverdin geometric isomer by means of HPLC/ESI–MS and NMR spectroscopy. Differentiation of the isomers by using fragmentation “in-source”

**DOI:** 10.1007/s00706-018-2161-7

**Published:** 2018-02-13

**Authors:** Rafał Frański, Błażej Gierczyk, Łukasz Popenda, Małgorzata Kasperkowiak, Tomasz Pędzinski

**Affiliations:** 1 Faculty of Chemistry, Adam Mickiewicz University, Umultowska 89B, 61-614 Poznan, Poland; 2 Adam Mickiewicz University, NanoBioMedical Centre, Umultowska 85, 61-614 Poznan, Poland; 3 Adam Mickiewicz University, Centre for Advanced Technologies, Umultowska 89c, 61-614 Poznan, Poland

**Keywords:** Biliverdin, Geometric isomer, Electrospray ionization, Mass spectrometry, NMR spectroscopy

## Abstract

**Abstract:**

A commercially available biliverdin sample was analyzed by means of HPLC/ESI–MS and NMR spectroscopy. It was been found that beside the main IXα 5*Z*,10*Z*,15*Z* isomer, the sample contains also the geometric isomer IXα 5*Z*,10*Z*,15*E.* It was also found the isomers behave differentially upon “in-source” fragmentation in negative ion mode (in contrast to the their behavior upon “in-source” fragmentation in positive ion mode and to their behavior upon MS/MS fragmentation in both modes): the relative abundances of deprotonated molecules and fragment ions are significantly different for both isomers, which can be used as an analytical tool to differentiate between the isomers.

**Graphical abstract:**

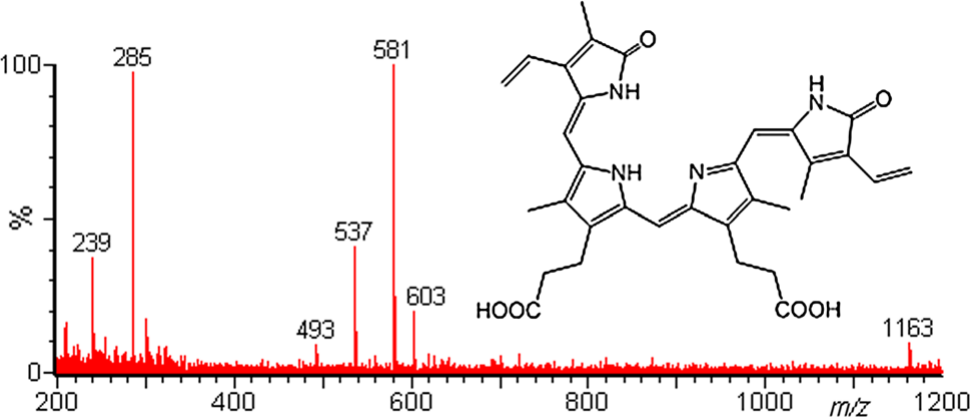

**Electronic supplementary material:**

The online version of this article (10.1007/s00706-018-2161-7) contains supplementary material, which is available to authorized users.

## Introduction

Biliverdin (BV) is a tetrapyrrolic pigment, a product of heme catabolism. Usually the term “biliverdin” refers to the main isomer of biliverdin, namely to IXα isomer. However, one should note that other positional isomers of biliverdin have been identified (e.g. XIIIα or IXδ) [1–11], and also besides the “natural” (5*Z*,10*Z*,15*Z*) isomer the geometric isomers (e.g. (5*Z*,10*Z*,15*E*), at the exocyclic double bonds are possible [12, 13]. Furthermore, the commercially available biliverdin may contain other positional isomers as well [14]. Therefore, we decided to check using HPLC/ESI–MS if biliverdin obtained from a commercial source as hydrochloride contains other isomers.

## Results and discussion

**Fig. 1:**
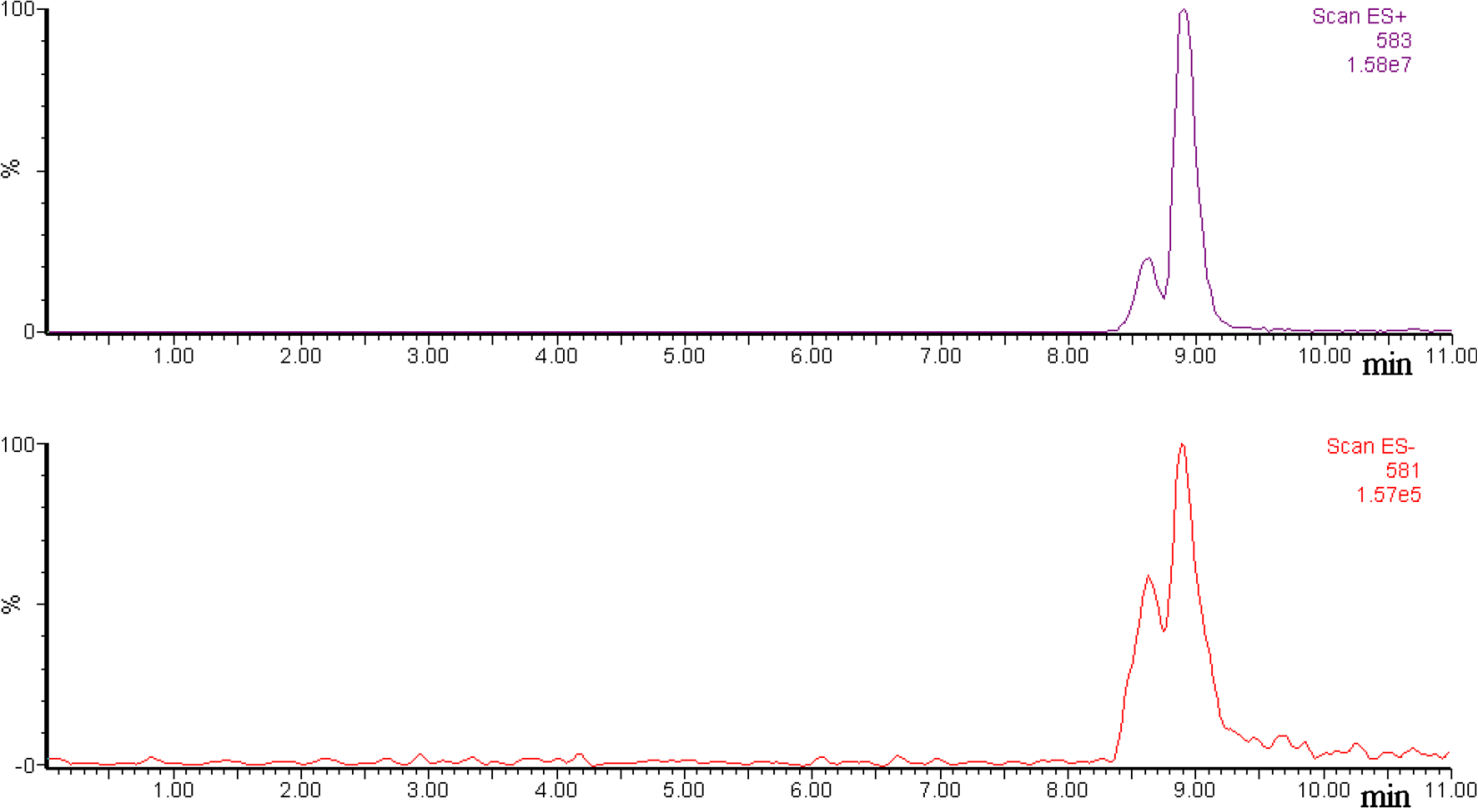
Single ion chromatograms of ions [BV + H]^+^ (top) and [BV−H]^−^ (bottom)

Figure 1 shows single ion chromatograms of ions [BV + H]^+^ and [BV−H]^−^ (*m*/*z* = 583 and 581) obtained upon HPLC/ESI–MS analyses.

As clearly shown in Fig. 1, for both ions two chromatographic peaks are obtained, thus the analyzed biliverdin sample contains two isomers. Taking into account the height of the peaks in the positive ion mode, the ratio of the main isomer to the minor isomer is about 5/1. A similar ratio was obtained by HPLC–UV/Vis analysis as shown in the Supplementary Material. As described further a similar ratio was also obtained by NMR spectroscopy. In the negative ion mode the peak ratio is different (Fig. 1), it indicates that the isomers may behave differentially upon ESI(−) conditions and this problem is discussed further in the text.

It can be taken for granted that the main isomer is IXα. However, the question is what is the minor isomer. To identify it by HPLC-ESI/MS we should have a respective isomer standards (to compare the retention times and ESI mass spectra). Fortunately, using NMR spectroscopy we were able to identify the minor isomer also as IXα, however, as *Z*,*Z*,*E*-isomer (obviously, the main isomer is *Z,Z,Z*), as discussed in detail below. The structures of both isomers (geometric isomers) are shown in Scheme 1.

**Figure:**
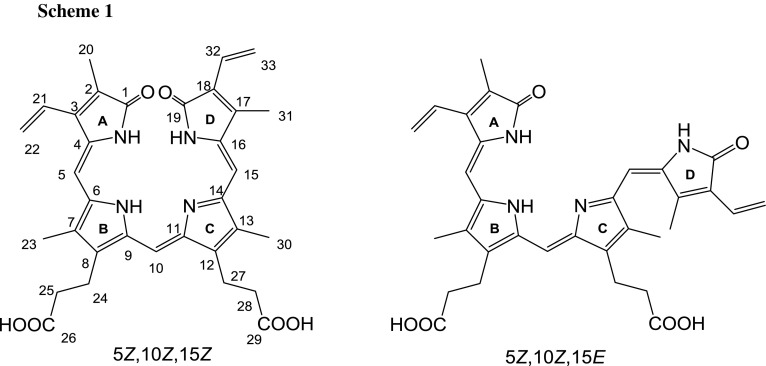

**Fig. 2:**
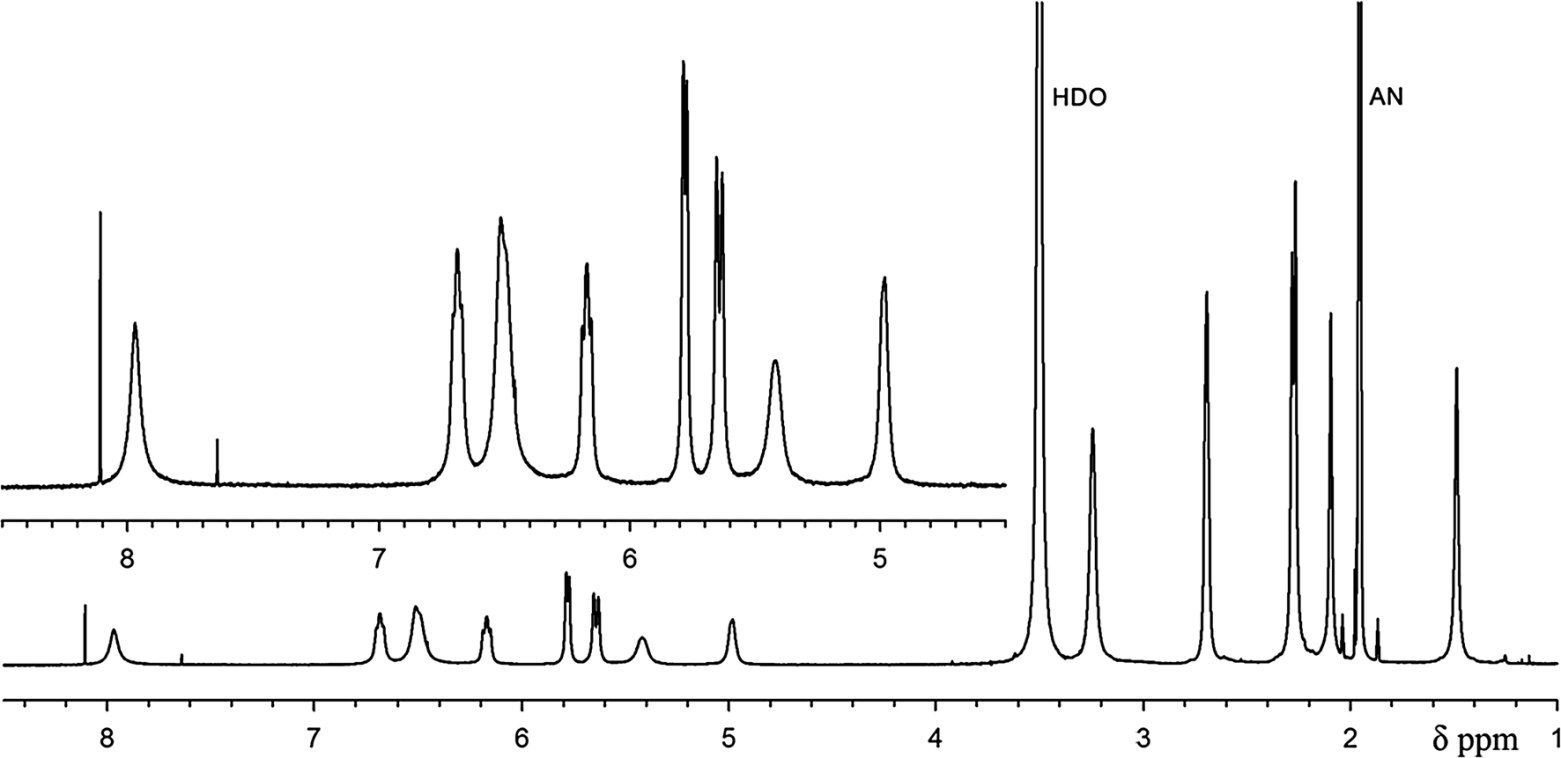
^1^H NMR spectrum of biliverdin sample in CD_3_CN/D_2_O (298 K)

**Fig. 3:**
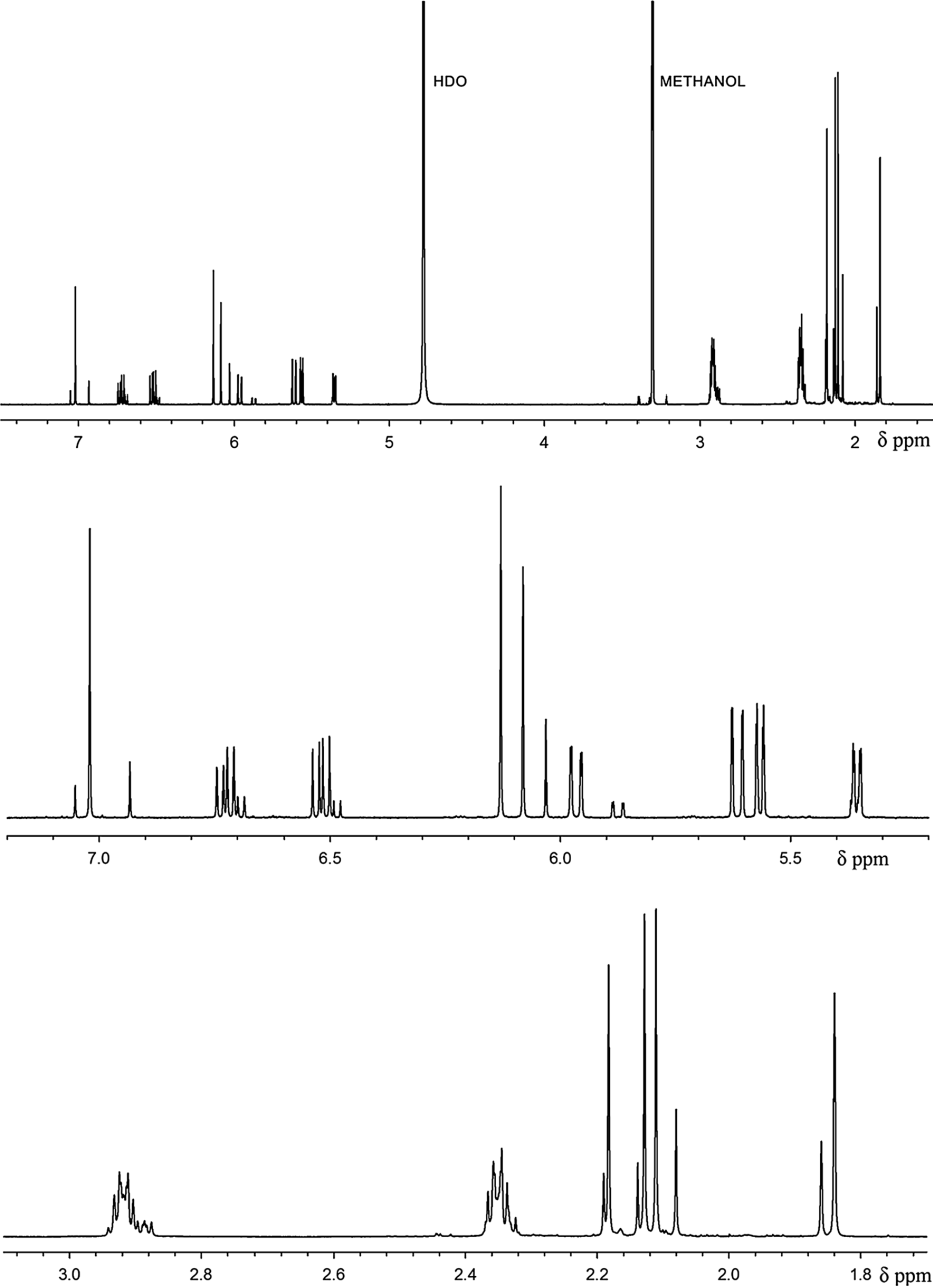
^1^H NMR spectrum of biliverdin sample in CD_3_OD/D_2_O/NaOD (298 K): full spectrum (top), selected regions (middle and bottom)

The ^1^H NMR spectrum of biliverdin hydrochloride, recorded in acetonitrile/water solution consists of one set of somewhat broadened signals, indicating a fast exchange in the NMR time scale (Fig. 2). However, in methanol/water/NaOD system (Fig. 3) two sets of signals were observed. The major ones were assigned to the *Z*,*Z*,*Z*-isomer of biliverdin, while the minor one to the *Z*,*Z*,*E*-isomer. The isomer ratio is about 5:1. The isomer structures were identified on the basis of a series of 1D selective NOE experiments. For the *Z*,*Z*,*Z*-isomer the enhancement of H-15 signal after irradiation of H-31 and H-30 was observed. For H-5 such an effect was recorded after irradiation of H-23 and H-21 or H-22 (vinyl group). In the *Z*,*Z*,*E*-isomer the NOE effects between H-31 and H-30 as well as H-30 and the vinyl group (H-32/33) were observed (Scheme 2). It is worth to add that an analogous geometric isomer has been described for biliverdin methyl ester [12, 13].

**Figure:**
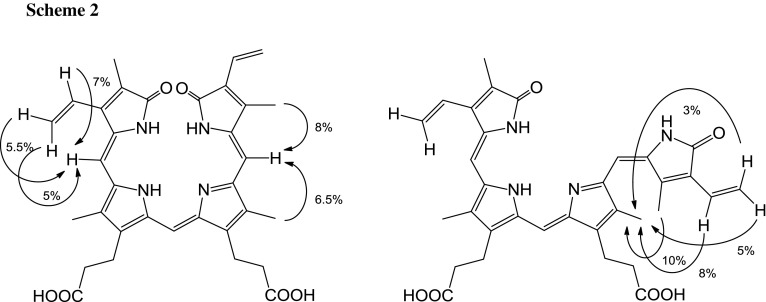

**Fig. 4:**
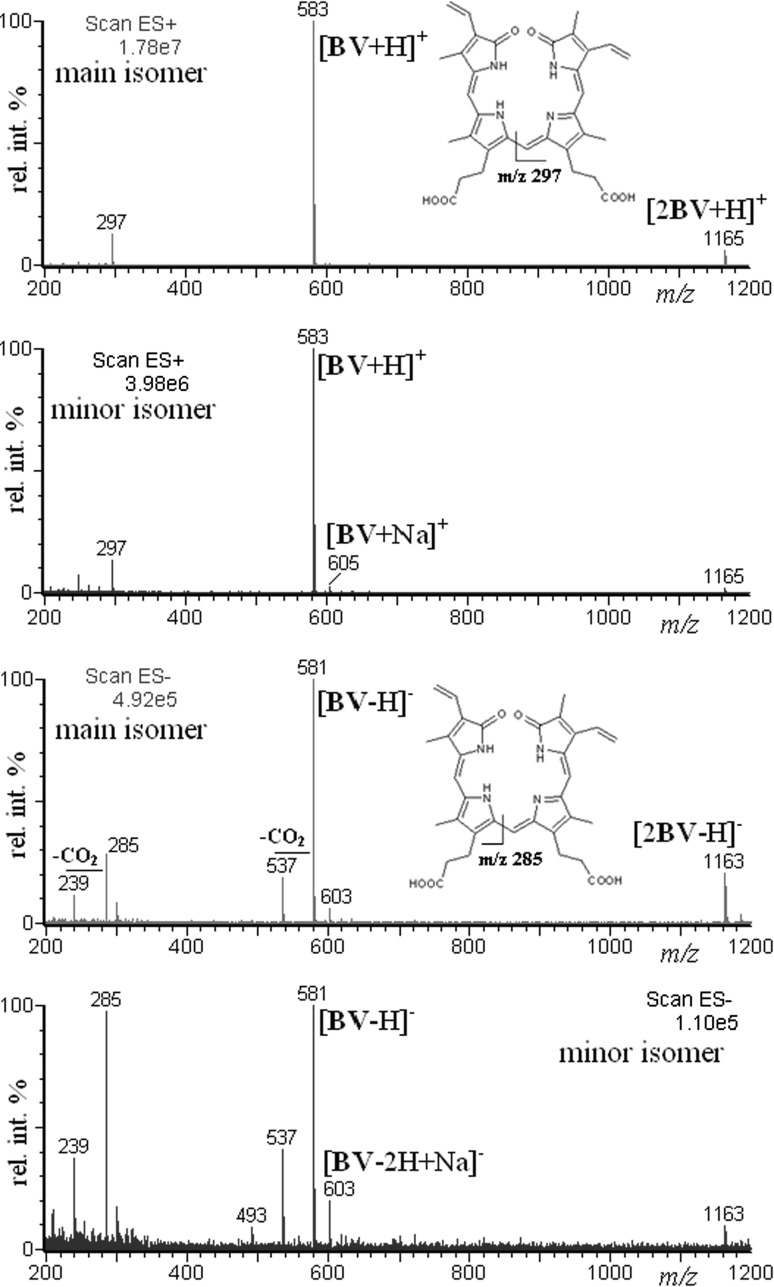
ESI mass spectra of bilverdin isomers obtained upon HPLC-ESI/MS analysis (CV = 50 V)

As mentioned earlier the isomers behave differently in ESI/MS conditions in the negative ion mode (Fig. 1). Figure 4 shows the ESI mass spectra of both isomers in the positive and negative ion mode at a cone voltage (CV) of 50 V as representative examples. The spectra obtained at other cone voltage values are shown in the Supplementary Material. The cone voltage has the most profound effect on the mass spectra obtained. Increase in this parameter leads to the so-called “in-source” fragmentation/dissociation (the pressure in this region is about 1 Pa), but a too low cone voltage may cause a decrease in sensitivity (less ions reach the high vacuum region). The observed decompositions of ions [BV+H]^+^ and [BV−H]^−^ in Fig. 4 are in good agreement with decomposition of this ions described elsewhere [15].

As clearly seen in Fig. 4, the spectra of both isomers obtained in the positive ion mode are quite similar. There is no difference in relative abundances of fragment ions. There are only minor differences in relative abundances of protonated dimers and sodium adducts. However, the spectra of both isomers obtained in the negative ion mode are different. Beside the differences in relative abundances of deprotonated dimers and sodium adducts, there is a significant difference in relative abundances of fragment ions (Fig. 4). It should be noted that in a few papers ESI/MS has been successfully applied for biliverdin analysis [16–21]. However, to the best of our knowledge, our finding is the first one which demonstrates the different ESI/MS behavior of two biliverdin isomers.

We also performed HPLC/ESI–MS/MS analysis of commercial biliverdin sample. However, the MS/MS spectra were very similar in both positive and negative ion mode. The results of HPLC/ESI–MS/MS analysis are presented in the Supplementary Material. In other words, there are differences in MS behavior of the isomers upon “in-source” fragmentation in negative ion mode, however there are no differences in MS behavior of the isomers upon MS/MS fragmentation (in collision chamber). The fragmentation of ions upon MS/MS experiments occurs later than that “in-source”. Therefore, it is reasonable to conclude that before the isomer ions reach the collision chamber, they isomerize to the identical structure. The fragmentation “in source” occurs almost immediately after the transfer of the ions from solution to the gas phase, thus the fragmentation reflects the structural differences of the biliverdin isomers present in solution.

The key question is why the differences upon fragmentation “in-source” of the isomers are in negative ion mode and not in positive ion mode. It is reasonable that in positive ion mode, protonation occurs at nitrogen atom of C ring. Such protonated biliverdin molecules may isomerize due to the resonance structures shown in Scheme 3.

**Figure:**
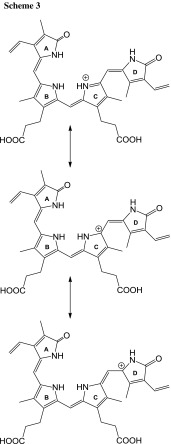

In negative ion mode, deprotonation of biliverdin molecule occurs at a carboxyl group. It is clear that the isomerization of deprotonated biliverdin molecules is not easy as the isomerization of protonated biliverdin molecules.

**Fig. 5:**
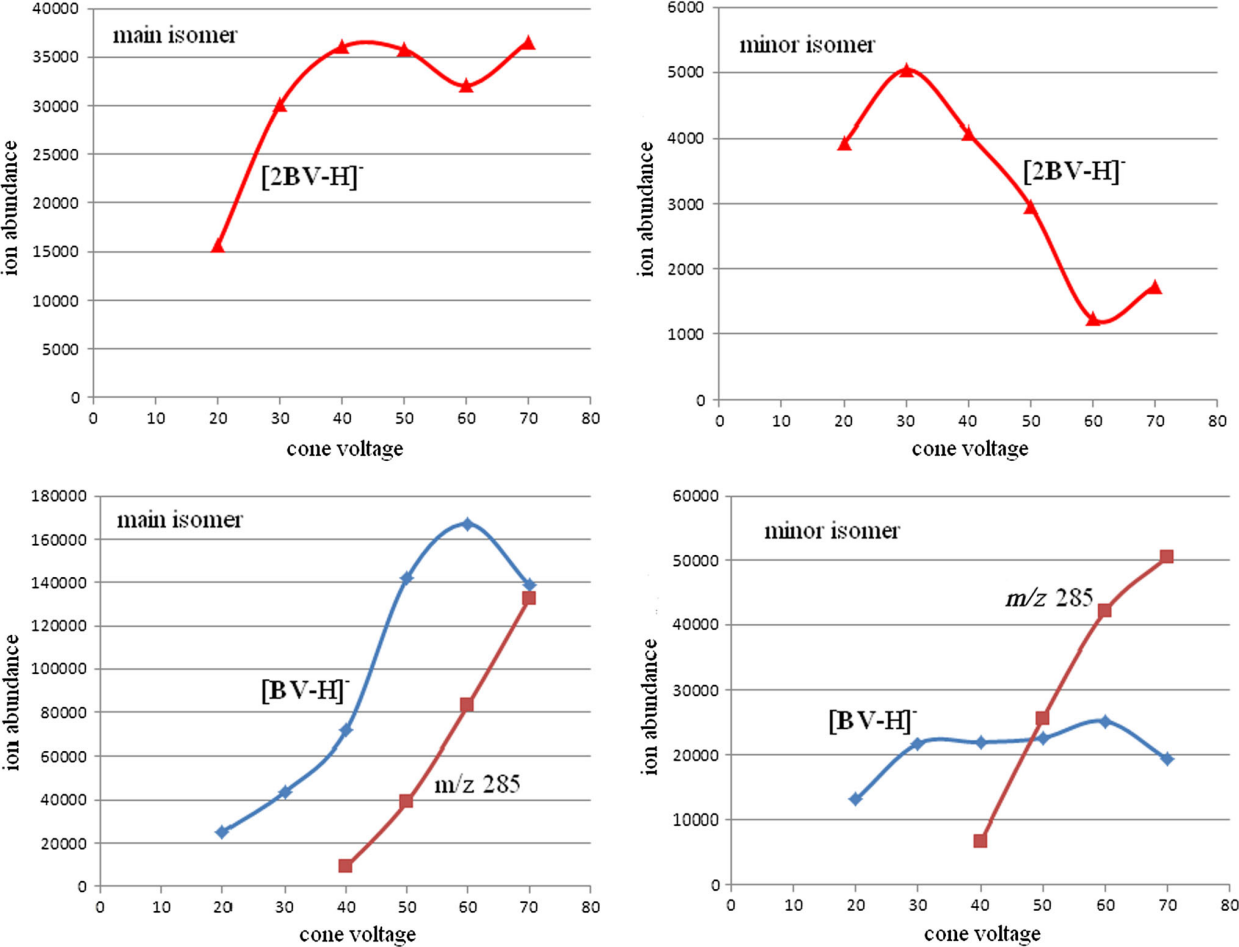
The breakdown plots of ions [2BV−H]^−^, [BV−H]^−^ and main fragment ion at *m*/*z* = 285, against cone voltage (V). The abundances of ions correspond to the respective peak areas obtained upon HPLC-ESI/MS analysis

To better understand the observed different behavior of the biliverdin isomers in the negative ion mode, we performed the breakdown plots of the respective ions, namely [2BV−H]^−^, [BV−H]^−^ and the main fragment ion at *m/z* = 285, against cone voltage. The breakdown plots are shown in Fig. 5.

As shown in Fig. 5 the gas phase stability of the [2BV−H]^−^ ion (deprotonated dimer) of main isomer is definitely higher than the gas phase stability of the [2BV−H]^−^ ion of the minor isomer (it is difficult to rationalize why at a cone voltage of 70 V we deal with an increase in [2BV−H]^−^ ion abundances for both isomers). The gas phase stability of the [BV−H]^−^ ion of the main isomer is also definitely higher than the gas phase stability of [BV−H]^−^ ion of the minor isomer. For both isomers decomposition of ions [BV−H]^−^ begins from the cone voltage 40 V (fragment ion at *m*/*z* = 285 is formed, Fig. 5). However, for the main isomer decomposition of [BV−H]^−^ ion is amply compensated by the sensitivity increase (at higher cone voltage more ions reach the high vacuum region). It is also worth adding that the decomposition of ions [BV−H]^−^ is compensated by the decomposition of ions [2BV−H]^−^. Taking into account the behavior of ions [2BV−H]^−^ (Fig. 5) it is clear that compensation of ion [BV−H]^−^ for the main isomer is more effective.

We have performed the breakdown plots of respective positive ions against cone voltage. As shown in the Supplementary Material, the breakdown plots for both isomers are similar.

## Conclusions

Using HPLC-ESI/MS and NMR spectroscopy, the minor isomer IXα 5*Z,*10*Z,*15*E* was found in a commercially available biliverdin sample (beside the main IXα 5*Z,*10*Z,*15*Z* isomer). The isomers behave differentially upon “in-source” fragmentation in the negative ion mode (in contrast to their behavior upon “in-source” fragmentation in the positive ion mode and to their behavior upon MS/MS fragmentation in both modes). It is difficult to rationalize why this very geometric isomer is present in the analyzed biliverdin sample. The geometric isomers are often formed as a result of exposure to light. However, our sample had been stored in the dark and frozen. It should be emphasized that our finding does not exclude such a commercial biliverdin sample from its use for scientific (e.g. analytical) purposes. Quite the opposite, this sample may be useful for analysis of both isomers, at least for semi-quantitative analysis.

## Experimental

Biliverdin (as hydrochloride) was obtained from Sigma-Aldrich (Poznań, Poland) and used without purification.

^1^H NMR spectra were recorded on Agilent DD2 800 spectrometer, operating at frequency 799.83 MHz. All spectra were measured at 298 K. The signal assignment has been made on the basis of 2D spectra (gCOSY, gHSQCAD, gHMBCAD) and 1D selective NOE measurements (mixing time 500 ms). Samples were prepared by dissolution of 5 mg of biliverdin hydrochloride in 0.7 cm^3^ of [^2^H]_4_-methanol, containing 10% of [^2^H]_2_-water and 0.05% of NaOD or in 0.7 cm^3^ of [^2^H]_3_-acetonitrile, containing 10% of [^2^H]_2_-water.

^1^H NMR (CD_3_CN/D_2_O): *δ* = 7.97 (bs, 1H, H-10), 6.69 (bm, 1H, H-21), 6.51 (bs, 1H, H-15?), 6.49 (bs, 1H, H-5?), 6.17 (bm, 1H, H-32), 5.78 (bd, 1H, *J* = 11.4 Hz, H-22), 5.64 (bd, 1H, *J* = 18.0 Hz, H-22), 5.41 (bs, 1H, H-33), 4.98 (bs, 1H, H-33), 3.23 (bs, 4H, H-24, H-27), 2.69 (bs, 4H, H-25, H-28), 2.28 (s, 3H, H-31?), 2.27 (s, 3H, H-23?), 2.10 (s, 3H, H-30), 1.48 (s, 3H, H-20) ppm.

^1^H NMR data assigned to the 5*Z*,10*Z*,15*Z*-isomer (main) (CD_3_OD/D_2_O/NaOD): *δ* = 7.02 (s, 1H, H-10), 6.73 (dd, 1H, *J* = 11.7, 17.9 Hz, H-21), 6.52 (dd, 1H, *J* = 11.6, 17.6 Hz, H-32), 6.13 (s, 1H, H-15), 6.08 (s, 1H, H-5), 5.97 (dd, 1H, *J* = 2.2, 17.6 Hz, H-33), 5.62 (dd, 1H, *J* = 1.6, 17.9 Hz, H-22), 5.57 (dd, 1H, *J* = 1.6, 11.7, H-22), 5.36 (dd, 1H, *J* = 2.2, 11.6, H-33), 2.92 (m, 4H, H-24, 27), 2.35 (m, 4H, H25, 28), 2.18 (s, 3H, H-31), 2.13 (s, 3H, H-23), 2.11 (s, 3H, H-30), 1.84 (s, 3H, H-20) ppm.

^1^H NMR data assigned to the 5*Z*,10*Z*,15*E* -isomer (minor) (CD_3_OD/D_2_O/NaOD): *δ* = 6.93 (s, 1H, H-10), 6.70 (dd, 1H, *J* = 11.7, 17.9 Hz, H-21), 6.50 (dd, 1H, *J* = 11.6, 17.8 Hz, H-32), 6.13 (s, 1H, H-15), 6.03 (s, 1H, H-5), 5.87 (dd, 1H, *J* = 2.0, 17.8 Hz, H-33), 5.62 (dd, 1H, *J* = 1.6, 17.9 Hz, H-22), 5.56 (dd, 1H, *J* = 1.6, 11.7, H-22), 5.37 (dd, 1H, *J* = 2.0, 11.6, H-33), 2.92 (m, 2H, H-24), 2.89 (m, 2H, H-27), 2.35 (m, 4H, H25, 28), 2.19 (s, 3H, H-31), 2.14 (s, 3H, H-23), 2.08 (s, 3H, H-30), 1.86 (s, 3H, H-20) ppm.

The HPLC-ESI/MS analyses were performed using a Waters model 2690 HPLC pump (Milford, MA, USA), a Waters/Micromass ZQ2000 mass spectrometer (single quadrupole type instrument equipped with electrospray ion source, Z-spray, Manchester, UK). The software used was MassLynx V3.5 (Manchester, UK). Using an autosampler, the sample solutions were injected onto the XBridge C18 column (3.5 µm, 100 × 2.1 mm i.d., Waters). The injection volume was 10 mm^3^ of biliverdin-containing solution at concentration 0.05 mg/cm^3^. The solutions were analyzed using linear gradient of CH_3_CN–H_2_O with a flow rate of 0.3 cm^3^/min. The gradient started from 0% CH_3_CN—95% H_2_O with 5% of a 10% solution of formic acid in water, reaching 95% CH_3_CN after 10 min, and the latter concentration was maintained for 10 min.

The mass spectra were recorded in the *m/z* range 200–1200, in positive and negative modes simultaneously (during the HPLC/ESI–MS analyses the mass spectrometer was switched in the fast mode between the positive and negative ion modes). The electrospray source potentials were: capillary 3 kV, lens 0.5 kV, extractor 4 V, and cone voltage 20–70 V (indicated in each mass spectrum shown). The source temperature was 120 °C and the desolvation temperature 300 °C. Nitrogen was used as the nebulizing and desolvation gas at the flow rates of 100 and 300 dm^3^/h, respectively.

## Electronic supplementary material

Below is the link to the electronic supplementary material.

Supplementary material 1 (DOC 412 kb)
