# Supplementary material for: Identification of a biliverdin geometric isomer by means of HPLC/ESI–MS and NMR spectroscopy. Differentiation of the isomers by using fragmentation “in-source”
Source: Monatsh Chem. 2018 Feb 13;149(6):995–1002. doi: 10.1007/s00706-018-2161-7 (PMC5972171; doi:10.1007/s00706-018-2161-7)
Supplement: Supplementary file 1 — Supplementary material 1 (DOC 412 kb) [file 706_2018_2161_MOESM1_ESM.doc]

**Identification of geometric isomer of biliverdin by HPLC-ESI/MS and NMR spectroscopy. Differentiation of the isomers by using fragmentation “in-source”. – *supplementary material***


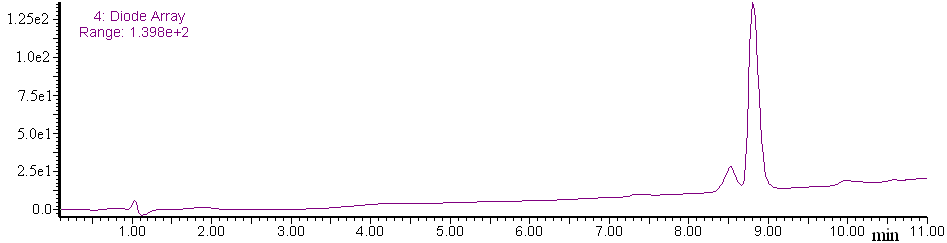


**Figure 1s.** UV/Vis chromatogram (200-600 nm) obtained upon analysis of biliverdin sample.


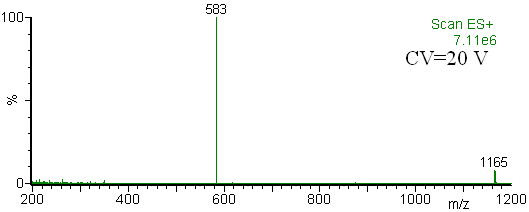


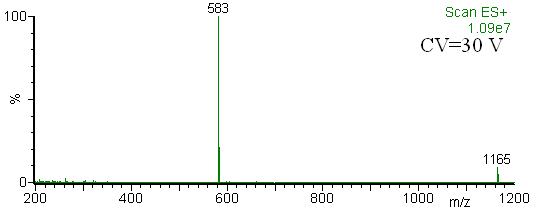


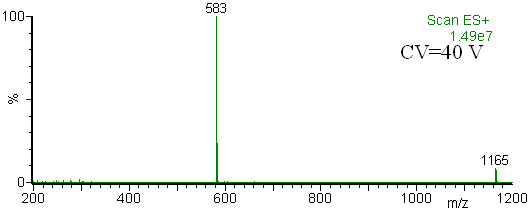


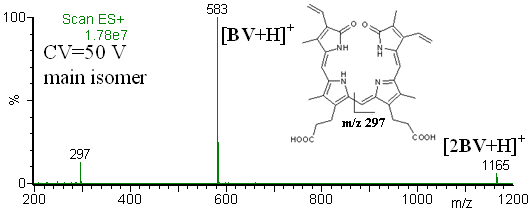


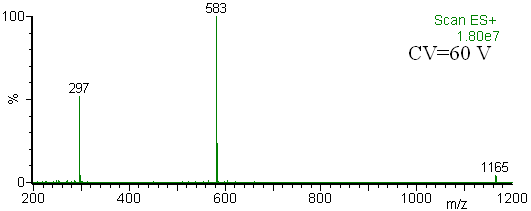


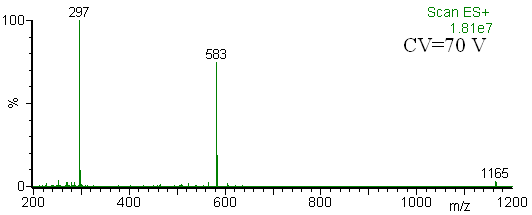


**Figure 2s.** ESI mass spectra of bilverdin main isomer obtained upon HPLC-ESI/MS analysis in positive ion mode.


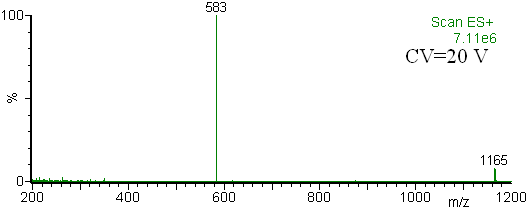


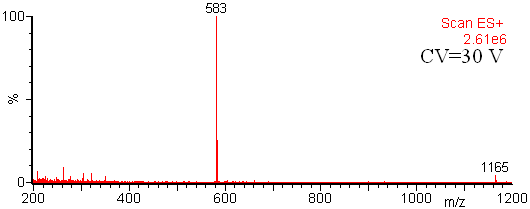


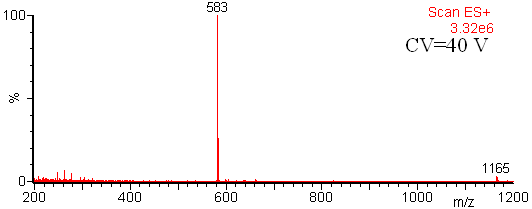


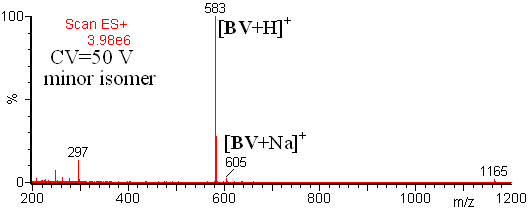


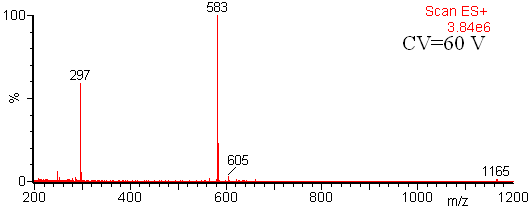


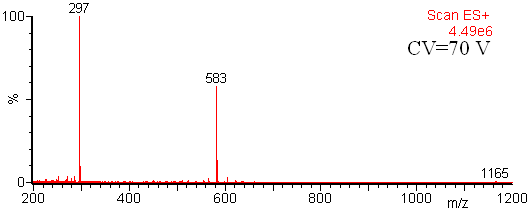


**Figure 3s.** ESI mass spectra of bilverdin minor isomer obtained upon HPLC-ESI/MS analysis in positive ion mode.


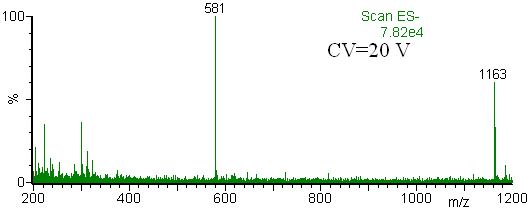


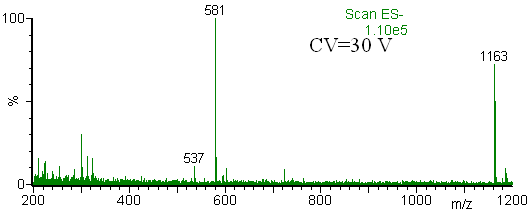


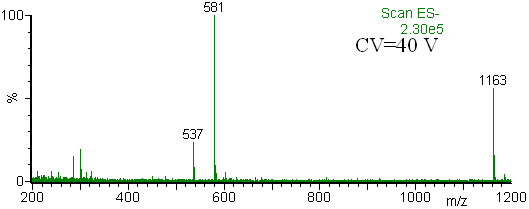


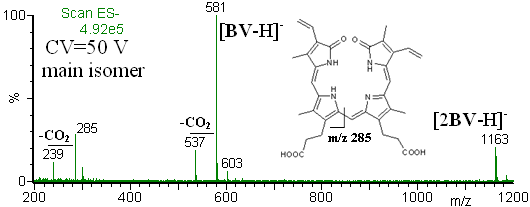


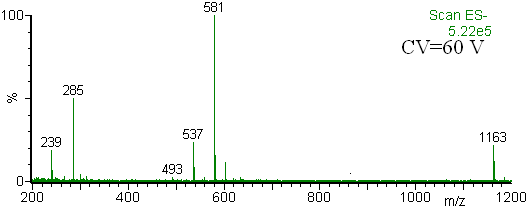


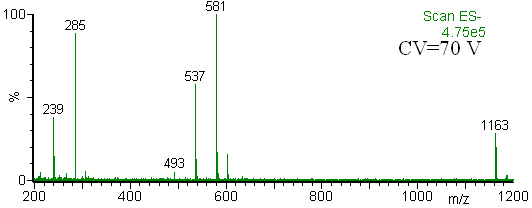


**Figure 4s.** ESI mass spectra of bilverdin main isomer obtained upon HPLC-ESI/MS analysis in negative ion mode.


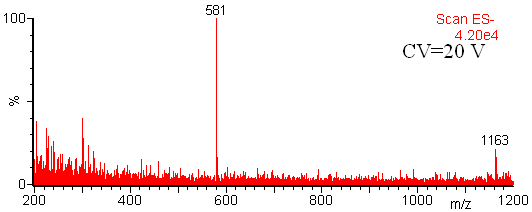


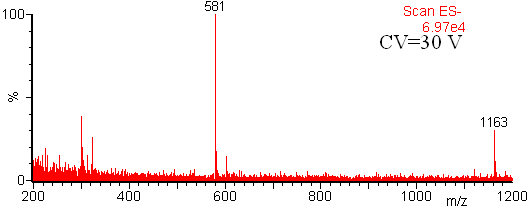


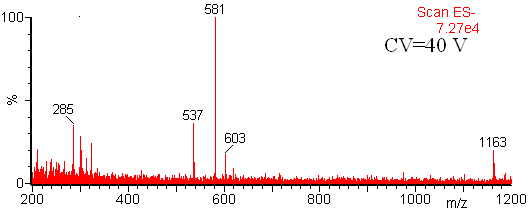


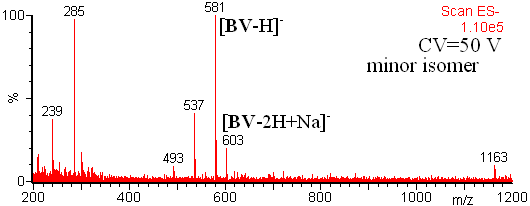


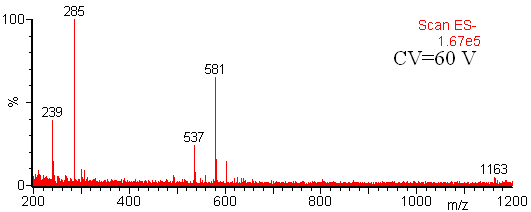


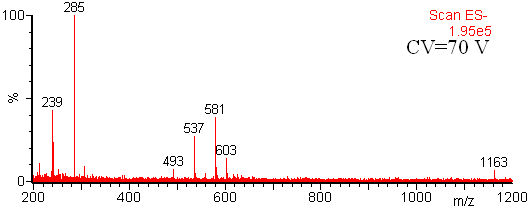


**Figure 5s.** ESI mass spectra of bilverdin minor isomer obtained upon HPLC-ESI/MS analysis in negative ion mode.

The HPLC/ESI-MS/MS analyses were performed using a UltiMate 3000 UHPLC System (Thermo Scientific) equipped with Kinetex C18 column (Phenomenex, 2.1 mm × 100 mm, 2.6 μm) and impact HD (ESI-QTOF) mass spectrometer (Bruker Daltonics). The injection volume was 2 μl and the elution flow rate 0.3 ml/min. The chromatographic separation was performed at 25 °C in gradient mode and the mobile phase was water with 0.1% formic acid (A) and acetonitrile with 0.1% formic acid (B). The gradient program was as follows: 0–0.5 min 5% B; 0.5–8 min from 5% to 90% B; 8–9.5 min 90% B; 9.5–14 min from 90% to 5% B; 14–16 min 5% B. The mass spectrometer was operated in positive and negative mode under the following optimized settings: capillary voltage 4.0 kV (for ESI+) and 3.4 kV (for ESI-); nebulizer pressure 1 bar; dry gas (nitrogen) temperature 200°C; dry gas flow rate 6 l/min. The applied collision energy, the most important parameter for MS/MS experiments, is indicated in each MS/MS spectrum caption. It has to be emphasized that HPLC/ESI-MS/MS analyses were performed at different collision energies and for both isomers the spectra were practically identical. Representative examples are shown at Figures 7s, 8s, 10s, 11s.

By using the above described conditions the HPLC-MS analysis were also performed in the full scan mode (respective total ion current chromatograms were obtained).


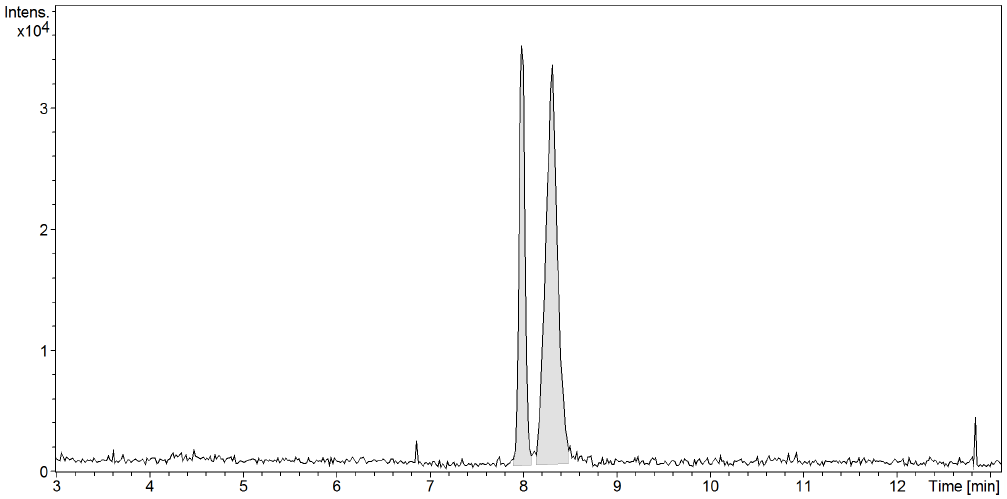


**Figure 6s.** Total ion current chromatogram obtained for biliverdin samples obtained on UltiMate 3000 UHPLC System (Thermo Scientific) coupled with impact HD (ESI-QTOF) mass spectrometer in negative ion mode.


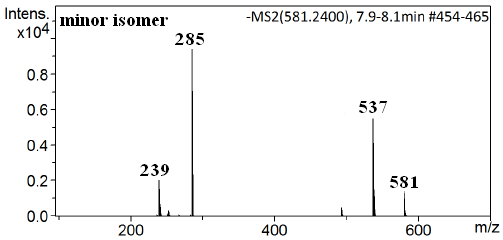

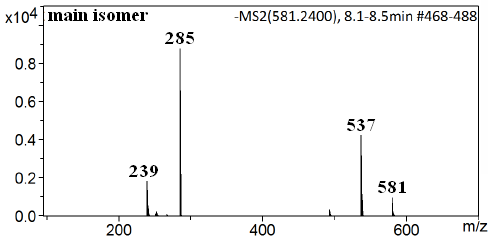


**Figure 7s.** ESI-MS/MS spectra of [**BV**-H]^-^ ions (collision energy 20 eV) obtained upon HPLC-ESI-MS/MS analyses.


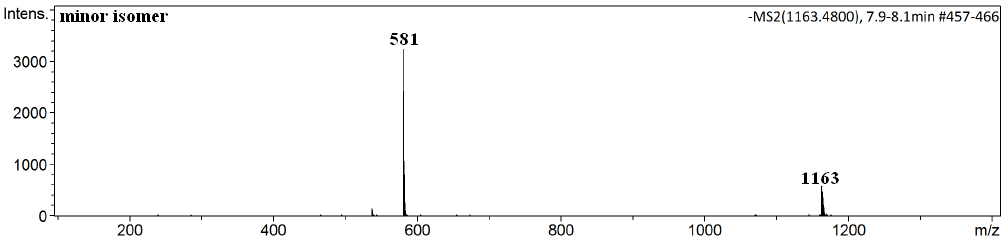


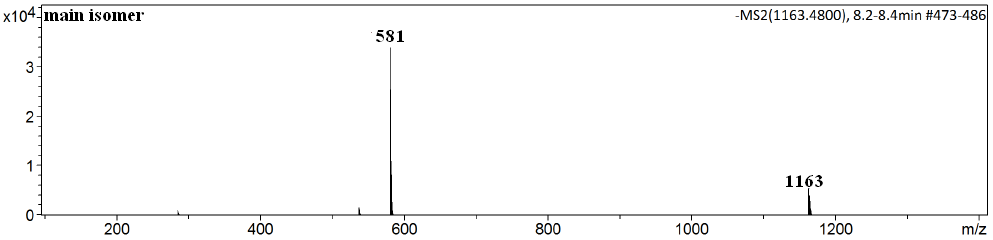


**Figure 8s.** ESI-MS/MS spectra of [2**BV**-H]^-^ ions (collision energy 20 eV) obtained upon HPLC-ESI-MS/MS analyses.


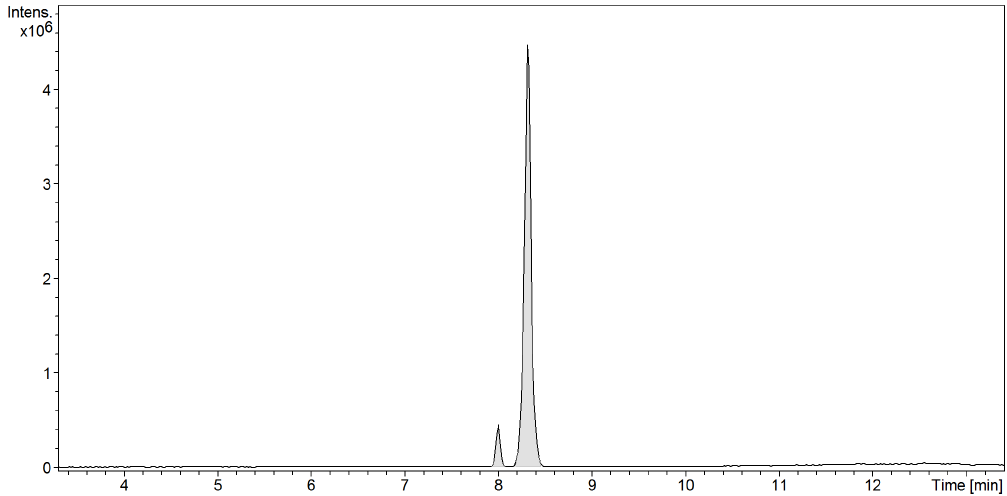


**Figure 9s.** Total ion current chromatogram obtained for biliverdin samples obtained on UltiMate 3000 UHPLC System (Thermo Scientific) coupled with impact HD (ESI-QTOF) mass spectrometer in positive ion mode.


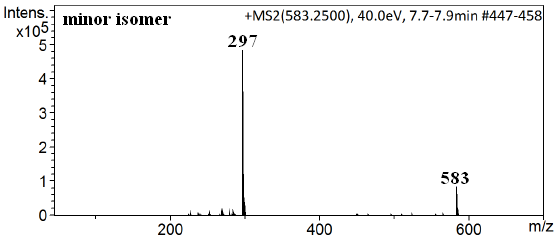

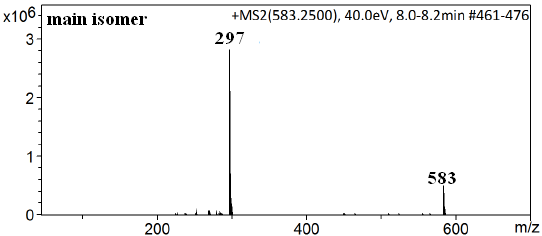


**Figure 10s.** ESI-MS/MS spectra of [**BV**+H]^+^ ions (collision energy 40 eV) obtained upon HPLC-ESI-MS/MS analyses.


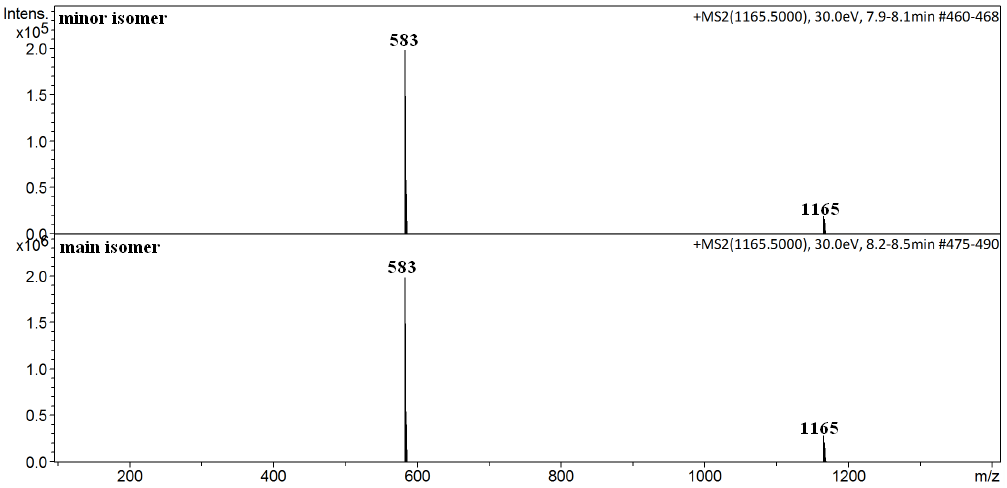


**Figure 11s.** ESI-MS/MS spectra of [2**BV**+H]^+^ ions (collision energy 30 eV) obtained upon HPLC-ESI-MS/MS analyses.


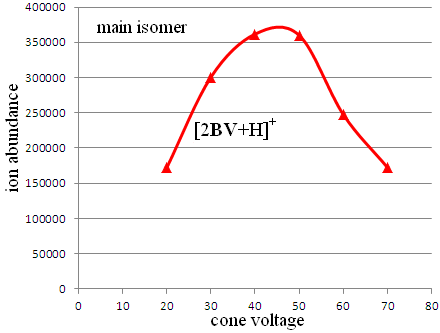

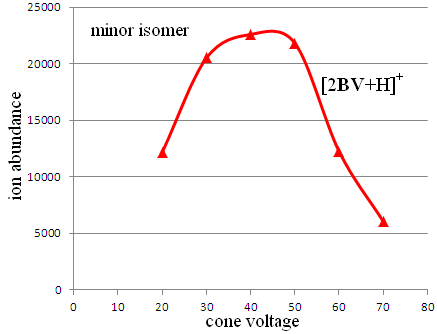


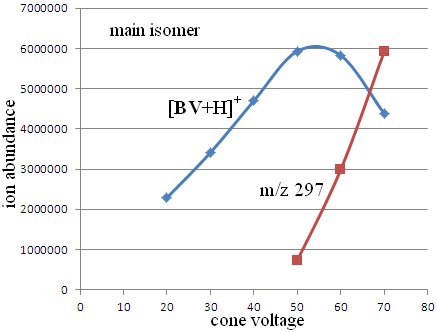

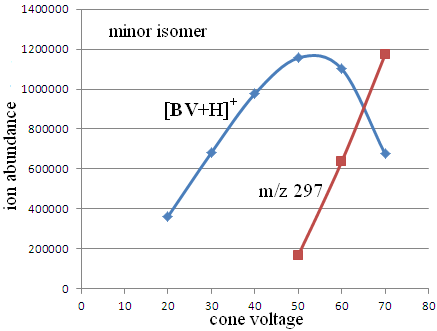


**Figure 12s.** The breakdown plots of ions [2**BV**+H]^+^, [**BV**+H]^+^ and fragment ion at m/z 297, against cone voltage. The abundances of ions correspond to the respective peak areas obtained upon HPLC-ESI/MS analysis.
